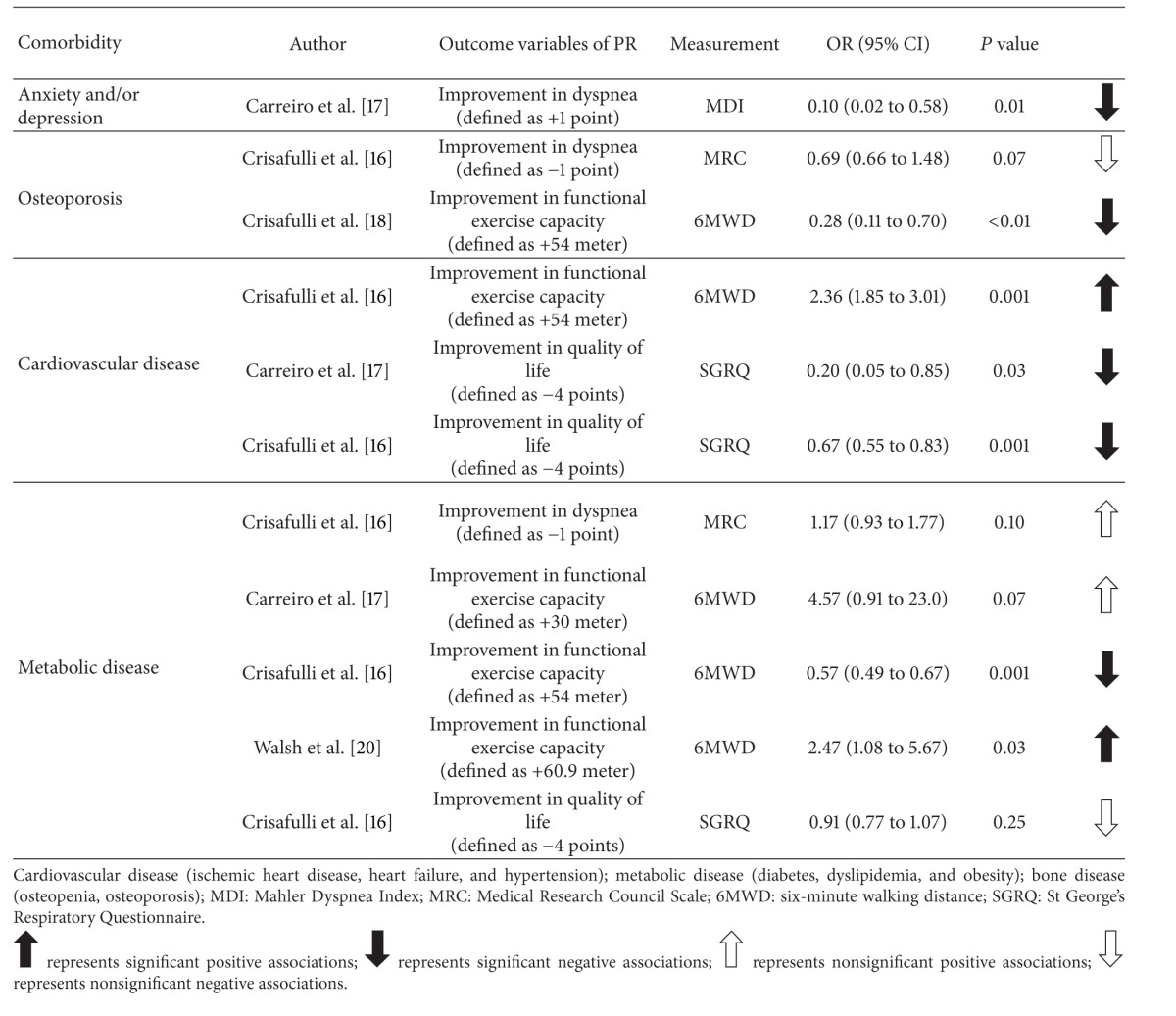# The Influence of Comorbidities on Outcomes of Pulmonary Rehabilitation Programs in Patients with COPD: A Systematic Review

**DOI:** 10.1155/2013/146148

**Published:** 2013-12-26

**Authors:** Miek Hornikx, Hans Van Remoortel, Heleen Demeyer, Carlos Augusto Marcal Camillo, Marc Decramer, Wim Janssens, Thierry Troosters

**Affiliations:** ^1^Department of Rehabilitation Sciences, Faculty of Kinesiology and Rehabilitation Sciences, KU Leuven, Belgium; ^2^Respiratory Division and Rehabilitation, University Hospital Gasthuisberg, Herestraat 49, 3000 Leuven, Belgium

## Abstract

*Introduction*. Chronic obstructive pulmonary disease (COPD) is associated with comorbidities such as cardiovascular disease, metabolic disease, osteoporosis, and anxiety and/or depression. Although pulmonary rehabilitation programs are proven to be beneficial in patients with COPD, it is unclear whether comorbidities influence pulmonary rehabilitation outcomes. The aim of the present review was to investigate to what extent the presence of comorbidities can affect pulmonary rehabilitation outcomes. *Methods*. The systematic literature search (Pubmed, EMBASE, and PEDro) resulted in 4 articles meeting the inclusion criteria. The odds ratios (95% confidence intervals) of the logistic regression analyses, with comorbidities as independent variables and pulmonary rehabilitation outcomes (dyspnea, functional exercise capacity, and quality of life) as dependent variables, were used for data extraction. *Results*. Patients with anxiety and/or depression less likely improve in dyspnea. Osteoporosis is associated with less improvements in functional exercise capacity, while cardiovascular disease does not seem to negatively impact on this outcome. Patients with cardiovascular comorbidity will experience less positive changes in quality of life. *Conclusion*. Evidence from literature suggests that comorbidities can have a negative influence on pulmonary rehabilitation outcomes. Screening for comorbidities in pulmonary rehabilitation settings seems useful to readdress the right patients for individually tailored pulmonary rehabilitation.

## 1. Introduction

Chronic obstructive pulmonary disease (COPD) is often described as a multicomponent syndrome, characterized by pulmonary and extrapulmonary consequences [1–3]. The pulmonary component of the disease is characterized by airflow limitation and chronic inflammation and typically gives rise to symptoms of cough, sputum production, and dyspnea [2]. The origin of the extrapulmonary consequences or comorbidities in COPD, which are prevalent alongside the disease spectrum [3–6], remains unclear. However, a lot of possible mechanisms have been suggested in literature. Systemic inflammation, inactivity, and deconditioning seem to have an important role in the development of these comorbidities [4, 7]. Cardiovascular disease, metabolic disease, osteoporosis, and anxiety and/or depression are the most frequently reported [1, 4, 6, 8, 9]. For all these conditions, rehabilitation and more specifically exercise training are indicated [10]. Pulmonary rehabilitation programs, generally consisting of both exercise training and strength training, are part of nonpharmacological interventions improving dyspnea, exercise capacity, quality of life, the amount of hospitalizations, and the recovery afterwards in patients with COPD [11–13]. Comorbidities increase the complexity of individual patients as evidenced by increased hospital admission rates and mortality [1–3]. It is, hence, plausible that conventional pulmonary rehabilitation may be more difficult in patients with comorbidities. On the other hand, the room for improvement may be larger in patients with COPD and comorbidity [14]. Literature does not provide clarity about the role of comorbidities on the success of pulmonary rehabilitation programs in patients with COPD. The aim of the present systematic review was to summarize the relevant literature on this topic, with the following research question: “Do comorbidities in COPD have an impact on outcomes of pulmonary rehabilitation programs”?

## 2. Material and Methods

This systematic review adhered where possible to the PRISMA (Preferred Reporting Items for Systematic Reviews and Meta-Analyses) statement on developing a systematic review [15].

### 2.1. Inclusion Criteria

Studies meeting the following criteria were included. (1) The study included stable patients with COPD (GOLDI-IV). (2) The patients with COPD should be screened in terms of comorbidities before starting pulmonary rehabilitation or comorbidities should be collected from medical records. (3) Comorbidities should include cardiovascular disease (ischemic heart disease, heart failure, and hypertension) and/or metabolic disease (diabetes, dyslipidemia, and obesity) and/or osteoporosis, and/or anxiety and/or depression. (4) The study predicted the outcomes of standard outpatient rehabilitation programs, that is, dyspnea, exercise capacity, or quality of life. Reviews were not selected, but references were hand-searched for relevant literature.

We did not report muscle weakness as a comorbidity in the present review as it can be assumed that muscle weakness is most likely present in those patients referred for pulmonary rehabilitation.

### 2.2. Search Strategy

A systematic electronic literature search was performed in Pubmed, PEDro (Physiotherapy Evidence Database), and EMBASE (Excerpta Medica dataBASE). The search terms we used included COPD, comorbidities or extrapulmonary comorbidities, and rehabilitation. In PEDro, the search was performed by indicating COPD, fitness training, and cardiothoracics in the proposed key terms. A more detailed description of the search strategies is depicted in Table 1. Reference manager 11 was applied to combine all the records from the three databases, to exclude duplicates and to provide information about the title and abstracts for abstract screening. The review team consisted of two reviewers (MH and HVR), who screened the title and abstract of the retrieved articles. Papers that met all in- and exclusion criteria were labeled as “1,” other articles were excluded and labeled as “0.” When disagreement occurred, MH and HVR re-evaluated the specific records, discussed them, and gave a final score in consensus. Additional articles were picked up by reviewing the reference list of relevant articles (hand-search). Articles which were labeled as “1,” were selected for full text assessment to check if they met the predetermined in- and exclusion criteria.

### 2.3. Data Extraction

From each article, we extracted the study design, the type of analysis and the disease severity. The content of the rehabilitation program described in each article was used. The specific comorbidities, their prevalence and the outcomes of pulmonary rehabilitation were extracted and the odds ratio (95% confidence interval (CI)) from each logistic regression analysis was retrieved to investigate the impact of comorbidities on outcomes of pulmonary rehabilitation. If the odds ratio was not calculated, but the author did report the *β*-coefficient (standard error (SE)) from the logistic regression analysis, we calculated the odds ratio (OR) with the formula: OR = *e* ^*β*^. The lower limit of the 95% CI of the odds ratio was then found based on *β* − 1.96∗SE and the upper limit of the 95% CI based on *β* + 1.96∗SE. When only the *β*-coefficient without SE was presented, the author was contacted to become the 95% CI of the OR. A significant OR < 1 indicates that the presence of the specific comorbidity leads to a lower chance of improving in the outcome investigated. With a significant OR being higher than 1, it is more likely that the presence of the comorbidity leads to improvement in the outcome of pulmonary rehabilitation.

## 3. Results

**Figure 1:**
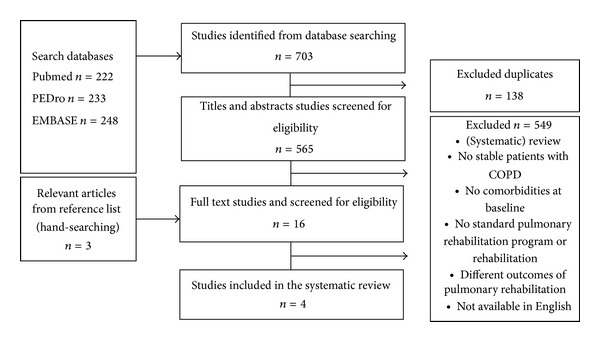
Flow chart of the results of the search strategies and study selection.

The systematic review resulted in 4 articles, involving a total of 3595 stable patients with COPD. The majority of patients (*n* = 2962) came from one study [16]. The flow chart of the results of the search strategies and the study selection are shown in Figure 1. Comorbidities were either retrieved from the medical record of the patients [16, 17] or were identified based on the Charlson Comorbidity Index [16, 18–20]. All articles reported comorbidities to be prevalent among patients with COPD, with a mean percentage of 70% of patients having one or more comorbidities. Pulmonary rehabilitation programs were supervised and involved either strength training or both strength and whole body exercise training. In 2 out of 4 articles, pulmonary rehabilitation programs were multidisciplinary [17, 18]. One article reported patients to be referred to standard outpatient rehabilitation, with no detailed description of the program [20]. None of the articles provided a description of the modifications of the goals of the program or the program content, taking into account the specific comorbidities. The pulmonary rehabilitation programs were beneficial in both patients with and without comorbidities. However, the improvements in dyspnea and health status were significantly less in patients with comorbidities, with patients diagnosed with more comorbidities achieving the least improvements [16]. Details about the comorbidities, the content of the pulmonary rehabilitation programs, the specific outcomes, and the response rates of the included articles can be found in Table 2.  Table 3 provides an overview of the obtained odds ratios (95% CIs) of the logistic regression analyses. More specifically, we concluded that patients with COPD and anxiety and/or depression have a 10 times higher chance of not reaching the minimal clinically important difference (MCID) in *symptoms of dyspnea* [17]. On the other hand, Crisafulli et al. [16] showed that having metabolic disease is associated with a higher chance of improving the symptoms of dyspnea and that osteoporosis is associated with a lower chance of improving the symptoms of dyspnea. However, these results were not significant. Improvements in *functional exercise capacity* were 4 times lower in patients with osteoporosis [18] and 2 times higher in patients with cardiovascular disease [16]. There was no consistency regarding the association between metabolic disease and functional exercise capacity, since 3 studies showed different results [16, 17, 20]. Patients with cardiovascular and metabolic disease will improve less in terms of *quality of life* [16, 17]. The relation between metabolic disease and quality of life did not show significance, while the association between cardiovascular disease and quality of life was found to be significant in the 2 studies [16, 17].

## 4. Discussion

The primary aim of our review was to summarize the literature concerning the impact of comorbidities on the outcomes of pulmonary rehabilitation. We found that the presence of comorbidities can have a negative influence on some outcomes of pulmonary rehabilitation. Overall, however, data are scarce. In detail, our review indicated that patients with symptoms of anxiety and/or depression are less likely to improve in dyspnea. In addition, patients with osteoporosis were found to improve less in terms of functional exercise capacity and patients with cardiovascular disease to improve more in functional exercise capacity. The findings on metabolic disease were inconsistent. Lastly, we found less positive changes in quality of life in patients with cardiovascular disease. A formal meta-analysis was not possible due to heterogeneity of the methods and the outcomes.

The present study contains several limitations. First of all, the majority of studies were retrospective, which could have impacted on the accuracy of collecting comorbidities. Another limitation was that we only included studies reporting odds ratios to show associations and studies providing data on improvements in pulmonary rehabilitation outcomes. Therefore, studies looking at comorbidities and outcomes in a different way were not withheld [19, 21–25]. The study of von Leupoldt et al. [25] confirmed our findings that anxiety and/or depression are associated with more symptoms of dyspnea. They showed that anxiety was positively associated with symptoms of dyspnea at rest (*β* = 0.18; *P* < 0.01), measured by the Borg scale before performing a 6MWD test. Both anxiety and depression were associated with more symptoms of dyspnea at baseline, measured by the Baseline Dyspnea Index (*β*_anxiety_ = −0.25; *P* < 0.001; *β*_depression_ = −0.35; *P* < 0.001) and after the 6MWD (*β*_anxiety_ = 0.15; *P* < 0.05; *β*_depression_ = 0.22; *P* < 0.05). Vanfleteren and colleagues [19] rejected the fact that cardiovascular disease is associated with higher levels of functional exercise capacity, by showing a negative association between ischemic heart disease and 6MWD (*β* = −0.11; *P* = 0.007). Mentz et al. [21] were not able to show significant different associations between either patients suffering from cardiovascular disease, that is, heart failure and COPD, or patients only diagnosed with COPD and outcomes of pulmonary rehabilitation. Trappenburg et al. [24] focused on maximal work rate, functional exercise capacity, and quality of life as dependent variables. Only the association between maximal work rate and symptoms of depression was significant, meaning that patients with COPD and depressive symptoms improve less in maximal work rate (*r* = −0.34; *P* = 0.008). The present review could not draw clear conclusions about the influence of metabolic disease on functional exercise capacity. However, Sava et al. [23] focused on obesity (BMI > 30 kg/m²) as a component of the metabolic disease and looked at its influence on the change in the 6MWD test. More specifically, they concluded that obese patients with COPD significantly improved in functional exercise capacity, but to the same extent as overweight patients and persons with a normal BMI. These findings are in line with the conclusions of Walsh et al. [20]. In another study on obesity, Ramachandran and colleagues [22] revealed that symptoms of dyspnea, investigated by the Chronic Respiratory Disease Questionnaire, were higher after pulmonary rehabilitation in patients with a BMI > 30 kg/m². In contrast, Crisafulli et al. [16] found patients with metabolic disease to have a higher chance of improving in symptoms of dyspnea, but these results were not significant. The last limitation was that the rehabilitation programs in the included studies were not adapted to the specific comorbidities. It remains unknown whether better adaptations of the rehabilitation programs to the comorbidities would have resulted in better outcomes.

Focusing on comorbidities in patients with COPD is of importance since they contribute to the overall severity of the disease and have a negative impact on patient's life expectancy [12, 26]. Considering this, there is an urgent need to address the role of comorbidities into the nonpharmacological treatment of patients with COPD. Although pulmonary rehabilitation programs are promoted by international guidelines to be an integral part of the management of COPD [11, 27], they largely focus on single diseases and do not take into account the comorbidities. Therefore, one must strive to develop guidelines for rehabilitation focusing on patients not on diseases so that treatment is in the individual's best interests [28]. Boyd et al. [29] confirm that comorbidities should receive more attention in patients with chronic diseases. In up to 55% of clinical trials in patients with COPD, patients with comorbidities are excluded [29]. This may explain why we only found 4 articles meeting our in-and exclusion criteria. Our findings can be supported by Patrick et al. [30], who investigated the effects of medical comorbidities on rehabilitation in geriatric patients. The focus of rehabilitation in the elderly addressed restoring functional independence and optimizing quality of life. They concluded that disability and cardiovascular, gastrointestinal, musculoskeletal, and endocrinal disease were significant negative predictors of rehabilitation efficiency in geriatric patients. On the other hand, not referring patients with comorbidities to pulmonary rehabilitation is surely not the way forward. All studies included in the current review demonstrate that patients with comorbidities can be trained with significant benefits. The real question is whether programs specifically tailored on comorbidities can improve these outcomes. Standard outpatient programs normally include exercise training as one of the most important components, which is proven to have a positive effect on cardiovascular disease, metabolic disease, and osteoporosis [26]. In order to achieve these benefits, patients with COPD have to train for 8–12 weeks, four times a week, at an intensity of 60–80% of maximal workload, during 20–30 minutes each session [31–33]. Based on the joint statement of the European Association for Cardiovascular Prevention and Rehabilitation, patients with ischemic heart disease and heart failure benefit from training sessions ranging from light (25–44% peak VO_2_, continuous training) to moderate (45–59% peak VO_2_, continuous training) to high (60–84% peak VO_2_, interval training) to very high intensity (≥85% peak VO_2_, interval training) to improve exercise capacity. The chosen intensity depends on the preserved ejection fraction and the exercise capacity of the patient during the pretraining period, measured by a cardiopulmonary exercise test [34]. Patients with arterial hypertension are instructed to train 30 minutes at moderate intensity (40–60% HRR), 5 times a week or 20 minutes at vigorous intensity (60–84% HRR), 3 times a week [35]. The guideline for patients with metabolic disease contains that patients have to perform 150 minutes of aerobic exercise, three times a week at moderate to vigorous intensity (40–60% VO_2_max) [35, 36]. Literature is available on the importance of exercise training in patients with osteoporosis. However, there is no clarity about the duration, intensity, or frequency of the training programs [37]. For patients with anxiety and/or depression, the advice is to train 3-4 times a week at moderate intensity (40% VO_2_max), during sessions of 20–30 minutes for a period of 8–14 weeks [38]. According to Fischer et al. [39] the intensity of the program being too high is the most often reported reason for drop-out in patients with COPD. In some patients, therefore, the guidance on training for comorbidities can overrule the guidance for training of the COPD related patients. A last point of attention relates to self-management as part of the disease management, which aims at changing the behavior of the patient by improving their problem solving skills. Clearly, these also need to be adapted to the comorbidities [40]. These literature findings defend our statement that comorbidities should be included into the management of patients with COPD, but that there is a need for cross-disease guidelines for rehabilitation. Training programs should be individually tailored and adapted to the specific comorbidity(ies) of the patient.

## 5. Conclusions

Comorbidities are prevalent in patients with COPD and they potentially have a negative effect on outcomes of standard pulmonary rehabilitation. More specifically, they could reduce the benefits in terms of dyspnea, functional exercise capacity, and quality of life. Based on the present review, we conclude that including patients with comorbidities in pulmonary rehabilitation programs is still reasonable as they improve with training. However, we should be aware that, without altering the program, response rates will be lower. An optimal treatment should therefore include a baseline assessment of comorbidities with a subsequent individually tailored pulmonary rehabilitation program.

## Figures and Tables

**Table 1:** Search strategy for the three different electronic databases.

Pubmed	(((((COPD) OR Chronic Obstructive Pulmonary Disease) OR Chronic Obstructive Pulmonary Disease [MeSH Terms])) AND (((Comorbidities) OR (Co-morbidities) OR Extrapulmonary comorbidities)) AND ((Rehabilitation) OR Rehabilitation [MeSH Terms]))

PEDro	((COPD) AND (Fitness training) AND (Cardiothoracics))

EMBASE	((“COPD” exp OR COPD) AND ((“Comorbidities”) OR (“Co-morbidities”) OR “extrapulmonary Comorbidities”) AND (“Rehabilitation” exp OR Rehabilitation))

**Table 2:** Detailed description of each individual article.

Author	Subjects	Analysis (study design)	Disease severity (mean FEV_1_ (% predicted))	Comorbidity(ies) investigated	Detailed prescription of pulmonary rehabilitation program	Outcome(s) study	Response rates in patients with comorbidities (% of patients reaching MCID)
Carreiro et al. [17]	*n* = 114	Retrospective (observational cross-sectional study)	46 ± 17	(i) Cardiovascular disease (68%) (ii) Metabolic disease (71%) (iii) Osteoporosis (11%) (iv) Anxiety and depression (21%)	(i) Period: 8 weeks (3*x*/week) (ii) Duration/session: 30–45 minutes (iii) Intensity endurance training: 80% PWR	(i) Dyspnea (MDI) (ii) Functional exercise capacity (6MWD) (iii) Quality of life (SGRQ)	(i) Dyspnea: 49% (ii) Functional exercise capacity: 63% (iii) Quality of life: 63%
Crisafulli et al. [16]	*n* = 2962	Retrospective (observational cohort study)	49 ± 15	(i) Cardiovascular disease (24%) (ii) Metabolic disease (62%) (iii) Osteoporosis (7%)	(i) Period: 20 sessions (3*x*/week) (ii) Duration/session: 3 hours (iii) Intensity endurance training: 70–80% PWR (iv) Strength training upper/lower body	(i) Dyspnea (MRC) (ii) Functional exercise capacity (6MWD) (iii) Quality of life (SGRQ)	(i) Dyspnea: 82% (ii) Functional exercise capacity: 60% (iii) Quality of life: 58%
Crisafulli et al. [18]	*n* = 316	Prospective (observational cohort study)	50 ± 14	(i) Cardiovascular disease (21%) (ii) Metabolic disease (56%) (iii) Osteoporosis (10%)	(i) Period: 21 sessions (3*x*/week) (ii) Duration/session: 3 hours (iii) Intensity endurance training: 70–80% PWR (iv) Strength training upper/lower body	(i) Dyspnea (MRC) (ii) Functional exercise capacity (6MWD) (iii) Quality of life (SGRQ)	(i) Dyspnea: 68% (ii) Functional exercise capacity: 60% (iii) Quality of life: 63%
Walsh et al. [20]	*n* = 203	Retrospective (observational cohort study)	53 ± 22	(i) Cardiovascular disease (32%) (ii) Metabolic disease (28%) (iii) Osteoporosis (12%)	No detailed information	Functional exercise capacity (6MWD)	No detailed information

Cardiovascular disease (ischemic heart disease, heart failure,and hypertension); metabolic disease (diabetes, dyslipidemia,and obesity); bone disease (osteopenia, osteoporosis); MCID: minimal clinically important difference; PWR: peak work rate; MDI: Mahler Dyspnea Index; 6MWD: six-minute walking distance; SGRQ: St George's Respiratory Questionnaire; MRC: Medical Research Council Scale.

**Table 3:** Overview of the logistic regression analyses performed in each article.